# Nursing Students’ Use of Recovery Stories of People with Mental Illness in Their Experiences: A Qualitative Study

**DOI:** 10.3390/nursrep12030060

**Published:** 2022-08-19

**Authors:** Ayako Yamashita, Takako Nakajima

**Affiliations:** 1 Department of Nursing, School of Health Sciences, Faculty of Medicine, Kagoshima University, Kagoshima 890-8544, Japan; 2 Department of Nursing, School of Health Science and Social Welfare, Kibi International University, Okayama 716-8508, Japan

**Keywords:** nursing student, mental illness, recovery story, qualitative study

## Abstract

This study clarified nursing students’ experiences of an educational program where they listened to the recovery stories of individuals with mental illness in a classroom setting. In this qualitative study, the program was delivered to third year nursing students in December 2019 (*n* = 62), after which they completed an anonymous free-response questionnaire. The responses were classified into seven clusters: understanding how patients perceive and appraise nursing care practices; interpreting experiences of disease realistically; deciphering patients’ histories based on their recovery stories; exploring ways to engage with patients based on knowledge of determinants of nursing care quality; finding ways to engage with patients grounded in respect; recognizing the importance of creating a therapeutic environment; gaining a sensitive understanding based on real-world stories. These clusters were grouped into “understanding the quality of nursing practices” and “gaining knowledge for application to nursing practices.”

## 1. Introduction

The burden of mental disorders is growing, having a significant effect on health and with major social, human rights, and economic consequences worldwide [1]. In Japan, the number of patients with a mental illness is steadily climbing; from 2002 to 2020, the national total increased from 2.58 to 5.03 million [2]. The country has taken specific policy measures to improve the local support for patients with psychiatric disorders as they transition back into the community, thereby expanding the comprehensive community care system to accommodate this population [3]. However, the average inpatient stay in Japanese psychiatric hospitals (298 days) ranks the longest worldwide, of which involuntary, medical-protective admissions (approximately 130,000 in 2020) or long-term stays lasting one year or longer (approximately 166,000 in 2020) account for most cases [4]. These circumstances place many demands on clinical psychiatric nursing. The nurses require high-level expert knowledge and skills in providing care in emergency and acute-phase treatments, community transition support for long-term inpatients, care for patients with physical comorbidities, and support for recovery as the patients transition to home healthcare. “Recovery” from a psychiatric illness is a process of changing one’s attitudes, values, emotions, goals, and skills to lead a satisfying life despite having a disease [5]. Evidence-based practice is fundamental to undergraduate and graduate nursing education and a way for the nursing discipline to minimize the theory-to-practice gap [6]. “Nursing practice for people requiring mental health care” is listed among the basic qualities and abilities required of nursing human resources in Japan’s Nursing Education Model Core Curriculum [7]. Several practitioners have written about the need to leverage the strengths of individuals with mental illness while supporting their recovery, thereby calling for efforts to enhance the providers’ understanding of the recovery concept [5] and incorporate practical and applied programs to train professional staff [8]. However, there are reports of negative and even stigmatizing attitudes toward patients with mental illness among nurses working in typical healthcare settings [9]. The attitudes of professionals influence the processes and quality of mental health care [10]. Negative attitudes and stigma among nurses interfere with their duty to support the recovery of patients with mental illness, impeding the provision of high-quality nursing care. Personal or professional exposure to people with mental illness effectively [11] reduces the stigmatization of this population among nursing students. Direct interactions with people with mental illness lead nursing students to recognize them as individuals, understand their life history, and mentally picture their recovery story, which deepens their understanding of their patients and helps them envision their recovery. Thus, direct exposure to people with mental illness is essential to nursing education aimed at recovery support.

Classroom programs using patients’ accounts of illness represent a teaching approach that is widely applied in the education of healthcare professionals. This approach—called narrative medicine—is an innovative and effective method for stimulating professional development and deepening patient understanding among healthcare providers [12]. Moreover, there is an increasing emphasis on, and commitment to, using patient narratives in nursing practice and nurse education [13].

Such lessons follow a participatory format; patient narratives are delivered by individuals with mental illness, who personally attend and address the class. Students are exposed to the disease in the form of a speech or story based on the reality and experiences of those affected by it (i.e., the narrative). Listening to patients’ illness narratives is a valuable opportunity to comprehend the experiences of mental illness and gain insights into nursing care.

Understanding the subjects’ life history is a component of nursing practice for which the importance in the recovery support of patients with psychiatric issues has been particularly highlighted [14]. Previous studies have revealed numerous learning effects on nursing students after listening to narratives from individuals with mental illnesses in Japan; these include a broadened understanding of life’s meaning and value [15] and a deepened understanding of disability [16]. Moreover, patient narratives have reduced prejudice toward individuals with mental illness and deepened students’ understanding of their daily hardships, frustrations, and strengths [17]. These findings imply that having students listen to the narrative of people with mental illness is a promising educational technique in the psychiatric nursing curriculum.

Educational programs that teach students to understand a patient’s disease experience and recovery story—the entire journey of the recovery process—and to internalize an abiding image of recovery are essential in supporting the recovery of individuals with psychiatric illness.

In Japan, the patients assigned to students in practical coursework in psychiatric nursing education are typically patients with chronic diseases under long-term hospitalization, which offer little connection to recovery models [18]. The content makes it difficult for instructors in psychiatric nursing courses to explain the inner world and psychiatric symptoms of patients with mental illness. Therefore, a commonly used lesson format is to have students listen to actual accounts from affected patients, which is a method for teaching them which nursing knowledge and skills are required for this population. The importance of nursing students understanding the narratives of people with mental illness must be recognized by both nursing education institutions and healthcare providers. These studies have reported learning outcomes achieved by nursing students after listening to the narratives of individuals with psychiatric illnesses, but none have attempted to validate the personal experiences of students in such lessons.

Therefore, clarifying the experiences of nursing students who have participated in an educational program is necessary. This involves listening to the recovery stories of individuals with mental illness in a classroom setting and offer insights into how recovery in this population can be targeted more precisely in nursing education.

Our efforts were guided by this research question: “How was the experience of nursing students participating in an educational program wherein they listened to the recovery stories of people with mental illness?”

## 2. Materials and Methods

### 2.1. Design

This exploratory study consisted of a cross-sectional survey using an anonymous free-response questionnaire, followed by a qualitative study of responses to extract key contents relevant to the study outcomes.

### 2.2. Sample and Setting

The study participants were a group of undergraduate nursing students enrolled at a single nursing university in Japan who were selected through convenience sampling. The participants comprised 62 third-year nursing students enrolled in the course Theories in Psychiatric Nursing. They participated in an educational program wherein they listened to the recovery stories of people with mental illness. Nursing universities are attended for four years in Japan. Usually, most students take the classroom component of psychiatric nursing in their second year and the first semester of their third year and the practical component from the second semester of their third year to the first semester of their fourth year.

### 2.3. Educational Program

Listening to recovery stories directly from people with mental illness is the defining feature of our classroom program. It was designed and executed by a two-person team: the class’s instructor and a part-time lecturer or psychiatric social worker representing the peer support center. A collaborative partnership presumes a coequal relationship. The professionals are self-aware of their expert knowledge but also acknowledge that the patient has unique knowledge, which is crucial to planning care programs and decision-making. For the guest speakers, we requested referrals from the peer support center specializing in mental health and welfare. We decided on two recommended peer supporters to attend the educational program as guest speakers. Table 1 presents the steps and activities of the lesson plan. The guest speakers and nursing students were given time to introduce their strengths to deepen their mutual understanding. The students prepared their self-introductions in advance. Each group of 7–8 students prepared a list of collective strengths by compiling each member’s strengths on a single poster board, which was used in a presentation on the day’s lesson. The guest speakers introduced their strengths using PowerPoint slides.

### 2.4. Data Collection

The data were collected in December 2019. The survey items were written based on the learning objectives shown in the course syllabus. Overall, seven questions were prepared, as shown below:

1. How did you feel about the guest’s experiential knowledge (i.e., how they mentally interpret their experiences)?
2. How did you reflect on aspects of therapeutic environments in psychiatric medicine?
3. How did you reflect on methods for forming trusting relationships with patients in psychiatric nursing?
4. How did you understand the significance of peer support in mental health and welfare?
5. How did the speakers’ stories help you understand patients from a recovery-oriented perspective?
6. How did the speakers’ stories help you understand patients from the strengths-based perspective?
7. How did the speakers’ stories help you think about how to be present as a supporter?

The responses were obtained anonymously using a free-response self-report paper questionnaire. The survey was uncompensated and took ~10 min to complete. After the program, the students were given a questionnaire and requested to participate in the research. The completed forms were retrieved from a collection box.

### 2.5. Analysis

The free-response texts concerning students’ experiences of listening to the recovery stories of people with mental illness were analyzed using the free software KH Coder (Ver. 3.0) [19]. A quantitative content analysis (or text mining) is a technique whereby researchers make qualitative interpretations of raw language by referencing the results of the quantitative analysis, thereby improving the data reliability [20]. KH Coder can reduce the “manual labor” of abstracting and presenting data, allowing the analysis to more explicitly target and eliminate bias caused by the analysts’ pet theories or the conceptualization of a research problem while still ensuring an objective and reliable analysis by manually abstracting and presenting data using a multivariate analysis. The first step of the analysis procedure was to convert the free-response texts to Excel data. Subsequently, the data were imported into KH Coder and morphologically analyzed and segmented into words using a morphological analyzer. Frequently occurring words were ascertained by creating a word frequency list to identify terms related to what nursing students learned while listening to the narratives of people with mental illness. The researchers checked the list for synonyms, near-synonyms, and polysemes; combined words covering the same meaning; and added their frequency counts in the final word frequency list. Further, a hierarchical cluster analysis (HCA; Ward’s method) was performed on those words that appeared five times or more in the data, with the number of clusters set automatically. Subsequently, based on the dendrogram generated by the HCA, two researchers checked for meaningful content in the data encompassing polysemous words that appeared in the HCA. Then, the researchers checked the hierarchical clustering of these terms, aggregated each cluster’s data according to its meaningful content, and decided on the cluster and theme names. To ensure that the analysis results were obtained with sufficient rigor [21], the researchers discussed them with two co-authors with sufficient experience in conducting qualitative research.

### 2.6. Ethical Considerations

The ethics review committee approved this study of the researchers’ institution. The peer supporters and the organization to which they belonged consented to participate in the study after receiving written and verbal explanations of its purpose. The lesson was not expressly held for this study. It has long been integrated within the Theories in Psychiatric Nursing course at the school. During the acquisition of informed consent, the students were informed that their feelings toward and participation in the study would have no bearing on their grades and that they would not suffer any disadvantage by not participating.

## 3. Results

Sixty-three questionnaires were distributed among third year nursing students at a nursing university. Of the 63 returned questionnaires, 62 were valid (an effective response rate of 98.4%).

### 3.1. Narrative-Based Learning Experiences

#### 3.1.1. Learning Outcomes Based on Most-Frequent Words

From 433 sentences, the text mining process calculated a token frequency of 9159 words (3352 words used) and a typical frequency of 774 words (586). Table 2 presents a list of the most frequently occurring words with a count of at least 10. The top three words were *omou* (to think, to feel; *n* = 149), *kiku* (to ask, to listen; *n* = 94), and *jibun* (myself, oneself; *n* = 87).

#### 3.1.2. Key Words Extracted from Free-Response Answers

The clusters and themes are denoted by square and angular brackets, respectively. The themes, clusters, and corresponding key words are presented in Table 3.

Theme 1: Understanding care quality in nursing practice.

Cluster 1—*Understanding how patients perceive and appraise nursing care practices*—shows how listening to the patient stories of being admitted and staying in a psychiatric hospital helped the students understand the realities of psychiatric nursing and how patients perceive nursing care quality.

*“I really got a good understanding by hearing them talk in detail about bad nursing care, their feelings, etc.”*

*(Nursing student 6)*

*“Hearing about his feelings when he entered the isolation room, there was so much anxiety and fear. I thought that we have to consider what kind of care we should provide (patients).”*

*(Nursing student 3)*

Theme 2: Gaining knowledge for application to nursing practice

Cluster 2—*Interpreting experiences of disease realistically*—shows how listening to guests’ personal accounts of mental illness helped the students appreciate the reality of patients’ experiences.

*“Hearing someone who experienced (mental illness) speak, I learned about the suffering of disease.”*

*(Nursing student 13)*

*“I was able to empathize after hearing about the care they had received in psychiatric wards and their progression to depression and schizophrenia.”*

*(Nursing student 21)*

Cluster 3—*Deciphering patients’ histories based on their recovery stories*—evidence of how listening to the guests’ accounts of their suffering and facing mental illness was an experience that helped them decipher the patients’ recovery stories.

*“I was really moved. I could tell they have faced up to and battled their illness for a long time.”*

*(Nursing student 4)*

*“I felt it was an amazingly valuable opportunity to hear a recovery story. Hearing their recovery stories, I was taken aback by the tough experience they had been put through. People who fall ill really understand themselves better than people around them and have a strong drive to live. That is what I learned.”*

*(Nursing student 17)*

Cluster 4—*Exploring ways to engage with patients based on knowledge of determinants of nursing care quality*—reflects how the guests’ narratives helped the nursing students understand how care affects the quality of psychiatric nursing and explore ways to interact with them to build supportive relationships.

*“I learned how good care and bad care each affect people receiving treatment.”*

*(Nursing student 10)*

*“I felt that patients’ spirits can be moved in a good direction by nurses’ actions, even trivial ones; it renewed my belief that I have to take responsibility for my own words and actions.”*

*(Nursing student 43)*

Cluster 5—*Finding ways to engage with patients grounded in respect*—shows how the guests’ narratives prompted the nursing students to respect the psychiatric patients and discover care approaches that acknowledge and support their thoughts and behaviors.

*“I learned that what is important is not rejecting the person, but patiently listening to them and having an attitude of understanding and respect.”*

*(Nursing student 1)*

*“I felt it was really important to think about the patient and care before acting as well as reaching out so that they know that I am by their side and accept them without rejection.”*

*(Nursing student 9)*

Cluster 6—*Recognizing the importance of creating a therapeutic environment*—shows how hearing about real conditions in psychiatric hospitals from the guests’ narratives helped the nursing students appreciate the importance of taking steps to keep the care environment a therapeutic one.

*“Patients are more likely to panic in a negative environment. It is important to create an environment that honors patients’ dignity.”*

*(Nursing student 25)*

*“I felt the importance of places where they can relax, people they can trust, and environments where they can do what they want.”*

*(Nursing student 17)*

Cluster 7—*Gaining sensitive understanding based on real-world stories*—reflects how listening to real accounts of disease in the guests’ narratives led the nurses to deeply empathize with them, thereby deepening their understanding of the patients.

*“I listened well to the speakers’ talk. I will never forget them.”*

*(Nursing student 29)*

*“This class really made me think.”*

*(Nursing student 7)*

## 4. Discussion

In this study, we clarified nursing students’ experiences of participating in an educational program wherein they listened to the recovery stories of individuals with mental illness in a classroom setting. Listening to the recovery stories of the guest speakers with mental illness prompted the nursing students to recognize the reality of the patients’ experiences of mental illness and to evaluate the quality of psychiatric nursing practices. The execution and use of evidence-based practices are important, high-priority components of providing recovery-oriented mental health services. Fostering autonomous motivation to support patient empowerment is one practice for which the effectiveness has been demonstrated [22]. Similarly, participating in training on assertive community treatment [23] and having opportunities for interacting with people who have gone through recovery [24] are effective measures that can improve the recovery orientation among nurses and other professionals engaged in psychiatric medicine and welfare services. Studies have highlighted the need for nurses to engage regularly with people with mental illnesses to elicit recovery [25]. The peer supporters who visited the classroom were people in recovery themselves; listening to their narratives, personal recovery stories, and experiences with medical and nursing care as psychiatric patients helped encourage recovery-oriented thinking among the nursing students in attendance.

We provided time during the program for those speaking—the peer supporters and nursing students—to introduce themselves and understand one another. The students could apply the strength model in the learning experience, specifically when introducing their strengths and hearing those of the peer supporters. The strength model [26] is an intervention technique for putting recovery-oriented care into practice in the mental health system [27]. Our program’s approach of focusing on strengths to foster an understanding of the patients promotes recovery orientation among nursing students. Dedicating time to reaching a mutual understanding between peer supporters and students built a partnership, which is an interpersonal dynamic necessary for building an effective treatment relationship.

The students demonstrated an understanding of how speakers perceive and judge the quality of different care practices, thereby gaining insights into psychiatric nursing practices. One cluster—understanding how patients perceive and appraise nursing practices—overlaps with the findings of the study [28] regarding a nurse’s attitude as a healthcare provider and ideals in medicine, nursing, and society. The written response of one student showed how she sympathized with a peer supporter’s experience with isolation treatment: “Hearing about his feelings when he entered the isolation room, there was so much anxiety and fear. I thought that we have to consider what kind of care we should provide [patients].” The opportunity to hear the experience of isolation and the realities of care at a psychiatric hospital directly from the guest speakers offered the students insights into how to care for people with behavioral restrictions. In our educational program, listening to the narratives of people who had received isolation treatment helped the nursing students empathize with their emotional experiences and better understand the quality of nursing care. This provided the students an opportunity to think about the experiences of patients who undergo forced or involuntary treatment and to consider how nursing care should be provided, e.g., issues related to environmental maintenance and managing patients’ anxiety and fear in psychiatric care. Moreover, the experience helped them to understand the realities of therapy in psychiatric medicine. Studies have reported that users of mental health services often feel anxious when they receive care, thereby feeling confused because they do not understand the situation [29]. Our lesson’s content allowed students to perceive people with mental illness as recipients of mental healthcare services and to evaluate the quality of care practices provided based on their narratives, thereby offering them insights into nursing practices. Nurse educators should choose speakers carefully to enhance the usefulness of the participatory lessons attended by guests affected by illness or their families. They must not necessarily confine the activities to personal narratives and should design lessons in collaboration with guests to take advantage of their central and first-hand knowledge of the illness. Our lesson plans take advantage of those with first-hand knowledge of recovery, adopting a format wherein active learning and collaboration with a peer support center were incorporated. Choosing a setting where peer supporters with mental illness and students could feel safe and relaxed during their interactions helped the students recognize the realities of their experience with illness and enhanced their understanding of their patients, thereby offering them insights into nursing practices.

Our study uncovered new knowledge, i.e., an educational program wherein nursing students listen to the recovery stories of people with mental illness deepens their understanding of patients as real people and how care practices influence the quality of mental health nursing. The experience prompts them to explore ways of interacting with them to build supportive relationships. This program can promote a recovery-oriented mindset among nursing students. Thus, recovery-oriented thinking can be promoted among nursing students by incorporating the strength model and collaborative partnership theory into classroom programs in psychiatric nursing education.

### Limitations

This study reports the results of a qualitative study of nursing students’ reflections on their participation in a classroom program wherein they listened to the recovery stories of individuals with mental illness. Since our questions focused on what the students learned from the patients’ experiential knowledge and recovery, their other thoughts may not be explicitly captured in their responses. When interpreting our results, readers should remember that the results may reflect population-specific attributes, as they were obtained via a cross-sectional survey at a single school and that the classroom program was the researchers’ own original design. The software used for data mining not only affects the reproducibility of the results, but also affected this study’s results. It should be included that use of this software could also present a limitation for this study in terms of not recognizing participants’ individual uses and interpretations of language.

## 5. Conclusions

This study identifies the need for nursing students to listen to the recovery stories of people with mental illness. Listening to the recovery stories of the guest speakers with mental illness prompted the nursing students to recognize the reality of the patients’ experiences of mental illness and to evaluate the quality of psychiatric nursing practices. Recovery-oriented thinking can be promoted among nursing students by incorporating the strength model and collaborative partnership theory into classroom programs in psychiatric nursing education.

## Figures and Tables

**Table 1:** Lesson plan of the educational program.

Steps	Activity	Time Required
1	Guest speakers introduce themselves.	10 min
2	Participants affirm joint agreement to maintain the classroom environment as a safe and relaxing space.	10 min
3	Guest speakers demonstrate “mood check” for self-monitoring.	10 min
4	Student groups and guest speakers introduce their respective strengths.	20 min
5	Participants share their impressions, opinions, and questions related to introductions.	10 min
6	Recovery stories presented by guest speakers.	20 min
7	Q&A session.	10 min

**Table 2:** List of most frequently occurring words (*n* ≥ 10).

Keywords (Japanese)	English Equivalent	Count	Keywords (Japanese)	English Equivalent	Count
*omou*	to think, to feel	149	*kankyou*	environment	17
*kiku*	to listen, to ask	94	*keiken*	experience	17
*jibun*	self, myself	87	*suki*	like	17
*kango*	nursing; care	76	*ohanashi*	chat; conversation	16
*wakaru*	to understand	63	*mukiau*	to face; to confront	16
*hanashi*	speech; chat	57	*aite*	partner	16
*kanja*	patient(s)	54	*hogo*	protection	16
*taisetsu*	important (serious)	54	*ki*	spirit; feeling(s)	15
*kangaeru*	to think, to consider	45	*gendou*	conduct (i.e., language or behavior)	15
*taiken*	experience (personal)	43	*seishin*	mind; spirit	15
*taiou*	care, respond to	42	*warui*	bad	14
*hito*	person	39	*hajimete*	for the first time	14
*shiru*	to know, to learn	38	*mitsukeru*	to find; to discover	13
*rikabarii*	recovery	32	*ima*	now	13
*kanjiru*	to feel	31	*hitei*	negative; reject(ion)	13
*nokoru*	to remain	29	*inshou*	impression	12
*kimochi*	feeling(s)	27	*kikeru*	to ask; to listen (potential form)	12
*jissai*	reality, actual	27	*tsuyoi*	strong	11
*hanasu*	to talk, to speak	27	*motsu*	to have; to hold	11
*rikai*	understanding, comprehension	26	*sutoorii*	story	10
*kokoro*	heart, mind	25	*ishi*	doctor	10
*ii/yoi*	good	23	*hitotsu-hitotsu*	one by one	10
*tsuyomi*	strength(s)	22	*manabu*	Learn	10
*koudou*	behavior, action(s)	22	*kyou*	today	10
*shien*	aid, assistance	21	*chiryou*	treatment, therapy	10
*taishou*	subject, target	19	*daiji*	important (great)	10
*byouki*	disease, illness	18	*hontou ni*	really	10

**Table 3:** A cluster analysis showing themes, clusters, and key words.

Theme	Cluster		Selected Words (English Translation)
Understanding the quality of care in nursing practice	1	Understanding how patients perceive and appraise nursing practices	One-by-one, behavior, conduct, spirit, remain, mind/heart, patient, nurse, impression, doctor, and care
Gaining knowledge for application on nursing practice	2	Realistically interpreting disease experiences	talk, ask/listen, experience, and actual
	3	Deciphering the histories of patients based on their recovery stories	face/confront, (one)self, think, understand, speak, like, strong, now, strength, comprehend, story, recovery, disease, experience, have, for the first time, person, mind/spirit, and know/learn
	4	Exploring methods for engaging with patients based on knowledge of determinants of nursing care quality	good and bad
	5	Finding methods for engaging with patients grounded in respect	subject, important/great, feeling, think, support, feel, partner, find, learn, reject, and important/serious
	6	Recognizing the importance of creating a therapeutic environment	treatment, environment, and protection
	7	Gaining sensitive understanding based on real-world stories	today, really, speech, and can ask/listen

## Data Availability

Not applicable.
